# Perceived discrimination and coping with substance use among Asian Americans during the COVID-19 pandemic: a cross-sectional analysis

**DOI:** 10.1186/s12889-025-21824-2

**Published:** 2025-02-20

**Authors:** Adrian Matias Bacong, Dale Dagar Maglalang, Janice Y. Tsoh, Anne Saw

**Affiliations:** 1 Division of Cardiovascular Medicine, Stanford University School of Medicine, Stanford, CA USA; 2 Stanford University Center for Asian Health Research and Education, Stanford, CA USA; 3 Asian American Research Center on Health, San Francisco, CA USA; 4 Silver School of Social Work, New York University, New York, NY USA; 5 Department of Psychiatry and Behavioral Sciences, University of California San Francisco, 675 18th Street, San Francisco, CA 94143 USA; 6 Department of Psychology, College of Science and Health, DePaul University, Chicago, IL USA

**Keywords:** Racial discrimination, Asian Americans, Alcohol and other drug use, COVID-19

## Abstract

**Background:**

Race/ethnicity-related discrimination against Asian Americans increased during the COVID-19 pandemic. Previous studies have found an association between discrimination and use of alcohol and other drugs (AOD) as a form of coping. In this study, we evaluate the association of stress from race/ethnicity-related discrimination and coping with tobacco, alcohol, or cannabis (marijuana or cannabidiol) among Asian Americans during the pandemic.

**Methods:**

We used data from Asian American participants of the Asian American and Native Hawaiian/Pacific Islander (AA & NH/PI) COVID-19 Needs Assessment Project (*n* = 3,159). We measured COVID-19 discrimination by racial/ethnic discrimination perceived as the greatest stressor, whether racial/ethnic discrimination impacted participants’ families, and perceived racial bias. Binary logistic regression examined the association between each AOD outcome, discrimination variables, and other COVID-19 stressors accounting for sociodemographic factors, physical and mental health, and survey medium.

**Results:**

Asian Americans used alcohol to cope with COVID-19 pandemic stressors (13.0%) followed by tobacco (4.3%) and cannabis (4.1%). About 24% of Asian Americans reported that racial/ethnic discrimination was the greatest source of stress. Racial/ethnic discrimination was only associated with cannabis use. However, COVID-19 stressors (aside from discrimination) were positively associated with all the AOD outcomes.

**Conclusions:**

Asian Americans’ AOD use for stress coping during the pandemic was prevalent. Perceived racial bias was associated with cannabis use, however other pandemic-induced stressors, not discriminatory in nature, were consistently associated with AOD use. Targeted research and policy efforts are warranted to address impacts from diverse stressors while tackling racism and substance use within Asian American communities to facilitate post-pandemic recovery.

**Supplementary Information:**

The online version contains supplementary material available at 10.1186/s12889-025-21824-2.

## Introduction

The COVID-19 pandemic led to an influx of reported discriminatory events towards Asian Americans (AA). Between March 2020 through December 2021, AA and Native Hawaiians/Pacific Islanders (NH/PI) reported 10,905 hate-related incidents to Stop Asian American and Pacific Islander (AAPI) Hate coalition [1]. While discrimination is often understood as direct verbal or physical attacks, experiencing discrimination can have complex deleterious effects.

Racial discrimination is associated with poor health behaviors and outcomes [2], including substance use [3, 4]. People of color may use substances as a form of coping from racism-induced stress. Thus, it is possible that with the rise of reported anti-Asian racism during the pandemic, AAs may increase their use of substances to cope [5, 6]. Studies found links between racism and substance use in the AA population [7, 8]. During the pandemic, 18.2% of U.S. adults increased or initiated their use of substances [9], albeit mostly related to alcohol use and cannabis use [10]. However, Hispanic/Latino people (36.9%) reported the highest prevalence of substance use while Non-Hispanic (NH) White people reported the lowest prevalence (14.3%) [9]. Studies examining substance use during the pandemic have noted heterogeneity in usage among AAs. One study found that AAs used drugs for fewer days compared to their racial counterparts [11]. Additionally, AAs had lower odds of increasing alcohol and cigarette use during the pandemic compared to NH White people [12]. However, a separate study comparing trends in alcohol use in 2020 versus the pre-pandemic period found that AAs had 1.3 times greater alcohol misuse in 2020 relative to NH White individuals [13].

Whereas these studies suggest that AAs may have heterogenous use of substances relative to other racial and ethnic groups during the pandemic, accounting for experiences of racism may influence substance use rates. Keum et al. [14] found that drinking to cope and depressive symptoms mediated the relationship between COVID-related racism and alcohol use severity among AA emerging adults [14]. National studies have also shown that experiencing racial discrimination and racial bias during the pandemic has been associated with greater psychological distress among AAs [15, 16]. For example, Shi et al. [16] found that nationally, experiencing racism during the pandemic was associated with 13.8 times higher odds of increased smoking among South Asian individuals. However, the association between experiencing racism or other forms of racial bias were not significantly associated with increased non-nicotine substance use among East Asian and Southeast Asian respondents. Alternatively, Zhou et al. [15] found that experiencing COVID-19 related racial/ethnic discrimination was associated with greater odds of depression, anxiety, and binge drinking among AAPI college students. The increased substance use among AAs during the pandemic is concerning. However, previous studies have not fully examined the motivations behind increased AOD substance use during the COVID-19 pandemic. It is possible that the association between racism and increased AOD substance use is due to substance use to cope with stress, including racial stress. Previous work has found that alcohol use to cope partially mediates the association between racial discrimination and alcohol related problems [17]. Additionally, racism may increase experiences of depression among AAs, leading to the use of alcohol and other drugs (AOD) as a coping mechanism [14]. While these findings provide preliminary evidence of the role of racism and racial discrimination on substance use and mental health, they only consider the singular role of racism on health, rather than the confluence of racism with other social and structural factors. Syndemic theory situates the experiences of racism and use of substances during the pandemic in the AA population. It argues that co-occurring health problems can interact, worsen, and be amplified in the context of and driven by deleterious structural and social conditions [18–20]. Using syndemic theory, Saw et al. [21] illustrated the compounding effects of health problems such as psychological distress and race-related stress experienced by AAs that was exacerbated during the pandemic driven by economic stress and historical and systemic racism targeting the AA population. Syndemic theory can also be employed to understand the use of substances in AAs during the pandemic by framing how different social factors may contribute to disparities in AOD use. In our study, we pinpoint racial discrimination as one of the primary contributors to AOD use to cope among AA populations. Similar to previous work [13–17], we argue that racism, both direct and indirect [22], contributes to AOD use to cope through worsening mental health. However, we acknowledge that there may be additional social and health factors that may also contribute to AOD use to cope with COVID-19 stressors. For example, other COVID-19 related stressors, such as the stress of isolation [23, 24] and socioeconomic stress [24, 25] may increase the likelihood of depression and potentially the use of AOD to cope. Additionally, other demographic factors, such as gender [26], age, marital status [27], and socioeconomic status [10, 28] may be associated with differences in AOD use to cope as well as confound the association between racial discrimination and AOD use to cope. Finally, differences in general AOD use by disaggregated AA group [29] as well as preexisting physical and mental health conditions [30] are important factors that could confound the association between racial discrimination and AOD use to cope.

**Table 1:** Weighted participant characteristics, the Asian American and Native Hawaiian/Pacific Islander COVID-19 Needs Assessment Project, *n* = 3,159

Variables	Total (*n* = 3,159)		
	% or Mean (SE)	Unweighted *N*	Weighted *N*
**Drug and Alcohol Use to Cope with COVID-19 Stressors**			
Tobacco Use	4.3	147	136.0
Alcohol Use	13.0	455	409.6
Marijuana/CBD Use	4.1	190	129.5
**Number of Substances Used**			
Zero Substances	83.4	2560	2634.0
1 Substances	12.5	435	395.1
2 Substances	3.5	135	109.8
3 Substances	0.6	29	20.2
**Combination of Substances Used**			
None	83.4	2560	2634.0
Alcohol Only	9.0	302	285.3
Tobacco Only	1.8	59	56.3
Marijuana/CBD Only	1.7	74	53.1
Alcohol and Tobacco	1.7	48	53.6
Alcohol and Marijuana/CBD	1.6	76	50.5
Tobacco and Marijuana	0.2	11	5.7
Alcohol, Tobacco, and Marijuana	0.6	29	20.2
**Impact and Experiences of Discrimination**			
Discrimination due to race/ethnicity greatest source of stress from the COVID-19 pandemic	24.5	1015	773.3
Facing Discrimination has impacted family’s life due to COVID-19 pandemic	23.5	909	73.8
CRBS Mean (Range: 1–5)	3.57 (0.02)		
**Number of COVID-19 Stressors Experienced (Range: 0–16)**	3.58 (0.06)		
**Demographic Variables**			
Age Category			
18–24 years old	13.1	1045	413.1
25–44 years old	43.1	1387	1360.7
45–64 years old	29.6	508	934.8
65 years old and older	14.3	219	540.5
Gender			
Man	48.4	1228	1530.2
Woman	50.5	1901	1596.0
Non-Binary or Other	1.0	30	32.7
Ethnicity			
Multiracial Asian	16.8	247	529.4
Chinese	18.6	699	588.8
Filipino	13.7	563	434.2
Indian	19.0	298	600.7
Vietnamese	8.5	427	267.5
Korean	6.3	411	197.5
Japanese	3.3	53	105.4
Pakistani	2.1	69	66.2
Other	9.5	138	299.8
Multiethnic Asian	2.2	254	69.6
Marital Status			
Married	59.6	1317	1883.4
Single	31.6	1673	999.2
Divorced, Separated, Widowed	8.7	169	276.3
**Acculturation Variables**			
Immigrant to the U.S.	62.8	1435	1983.8
Used English for Survey	85.1	2772	2689.3
**Socioeconomic Factors**			
Educational Attainment			
Less than High School	9.2	93	290.4
High School Graduate/GED	9.6	373	303.5
Some College	14.3	996	453.2
Bachelor’s Degree	33.9	1040	1069.3
Graduate Degree or More	33.0	657	1042.6
Household Income			
Less than $25,000	13.9	617	439.9
$25,000 - $34,999	4.7	263	147.8
$35,000 - $49,999	8.1	372	256.9
$50,000 - $74,999	14.1	502	445.6
$75,000 - $99,999	12.5	405	393.3
$100,000 or more	46.7	1000	1475.5
**Preexisting/Current Health Conditions**			
No Chronic or Mental Health Conditions	48.9	1687	1545.5
Chronic Condition Only	26.2	582	826.2
Mental Health Condition Only	14.0	596	442.2
Has Both Chronic and Mental Health condition	10.9	294	345.2
Survey Medium			
Community Organization	62.1	2097	1963.1
Panel	37.9	1062	1195.9

Note. CBD = Cannabidiol, CRBS = Coronavirus Racial Bias Scale, GED = General Educational Development, equivalent to a high school diploma

Total sum of the weighted percentages across mutually exclusive categories may not be exactly 100.0 due to rounding. Total sum of weighted n’s across mutually exclusive categories may not sum to exactly 3159 due to rounding

**Table 2:** Correlation matrix of discrimination, COVID-19 stressors, and drug and alcohol use, the Asian American and Native Hawaiian/Pacific Islander COVID-19 Needs Assessment Project (*n* = 3,159)

	Discrimination Stress	Discrimination Impacted Family	CRBS Mean	Total # of Stressors (No Discrimination)	Tobacco Use	Alcohol Use	Marijuana/CBD Use
Discrimination Stress	1						
Discrimination Impacted Family	0.47***	1					
CRBS Mean	0.47***	0.40***	1				
Total # of Stressors (No Discrimination)	0.38***	0.26***	0.32***	1			
Tobacco Use	0.03	0.07***	0.04	0.11***	1		
Alcohol Use	0.13***	0.11***	0.14***	0.20***	0.24***	1	
Marijuana/CBD Use	0.11***	0.10***	0.14***	0.15***	0.20***	0.29***	1

Note. CRBS = Coronavirus Racial Bias Scale, CBD = Cannabidiol, * *p* <.05, ** *p* <.01, *** *p* <.001

There is a paucity of data on the use of substances in the AA population since the pandemic’s onset. With the known proliferation of reported experiences of racial discrimination by AAs, it is crucial to understand the effects of discrimination on substance use during the pandemic. The current study: (1) evaluates the proportion of race/ethnicity-related discrimination and alcohol and other drug (AOD) substances used to cope, and (2) examines the association of stress because of discrimination related to race/ethnicity and other stressors and coping with AOD among AAs.

**Table 3:** Weighted multivariable logistic regression of tobacco use to cope with COVID-19 stressors on stress and impact of discrimination, the Asian American and Native Hawaiian/Pacific Islander COVID-19 Needs Assessment Project, *n* = 3,159

Variables	Model 1			Model 2			Model 3			
	OR	95% CI	*P* - value	OR	95% CI	*P* - value	OR	95% CI	*P* - value	
**Exposure and Impact of Discrimination and other non-discrimination-based stressors**										
Racial/ethnic discrimination was greatest source of stress during the COVID-19 Pandemic (No ref.)	0.94	0.47–1.89	0.87	0.71	0.35–1.42	0.33	1.20	0.58–2.47	0.62	
Racial/ethnic discrimination impacted family (No ref.)	1.85	1.02–3.36	0.04	1.72	0.94–3.15	0.08	1.53	0.86–2.71	0.15	
Coronavirus Racial Bias Scale Mean Score	1.15	0.71–1.84	0.57	1.06	0.66–1.72	0.81	1.03	0.57–1.85	0.93	
Sum of COVID Stressors, Except Discrimination				1.17	1.08–1.26	< 0.001	1.12	1.01–1.25	0.04	
**Demographic Factors**										
Age Category (65 years old ref.)										
18–24 years old							1.44	0.26–7.90	0.68	
25–44 years old							3.31	0.67–16.32	0.14	
45–64 years old							1.25	0.25–6.38	0.79	
Gender (Man ref.)										
Woman							0.41	0.25–0.65	< 0.001	
Non-Binary, Trans, or Other							0.25	0.02–2.46	0.23	
Race and Ethnicity (Multiracial Asian ref.)										
Chinese							0.60	0.25–1.44	0.26	
Filipino							1.13	0.50–2.52	0.77	
Indian							1.79	0.80–3.98	0.15	
Vietnamese							0.81	0.31–2.11	0.66	
Korean							1.75	0.73–4.19	0.21	
Japanese							3.43	0.94–12.53	0.06	
Pakistani							4.42	1.31–14.89	0.02	
Other Asian Ethnicity							0.64	0.17–2.43	0.51	
Multiple Asian Ethnicities							0.60	0.17–2.17	0.43	
Marital Status (Married or Living with Partner ref.)										
Single							0.82	0.44–1.56	0.58	
Divorced, Separated, or Widowed							3.16	1.26–7.91	0.01	
**Acculturative Factors**										
Immigrant to U.S. (Non-immigrant ref.)							0.66	0.40–1.11	0.12	
Used English in Interview (Other Language ref.)							0.73	0.34–1.60	0.44	
**Socioeconomic Factors**										
Educational Attainment (Less than High School ref.)										
High school graduate/GED							6.52	1.21–35.23	0.03	
Some college or associate degree							4.98	0.98–25.18	0.05	
Bachelor’s degree							2.94	0.57–15.30	0.20	
Graduate degree							1.26	0.23–6.97	0.89	
Household Income (< $25,000 ref.)										
$25,000 to $34,999							2.76	1.05–7.25	0.04	
$35,000 to $49,999							1.48	0.59–3.70	0.41	
$50,000 to $74,999							1.67	0.66–4.21	0.28	
$75,000 to $99,999							0.71	0.24–2.08	0.53	
$100,000 or more							2.58	0.96–6.89	0.06	
**Health Factors**										
Preexisting/Current Health Conditions (No Chronic or Mental Conditions ref.)										
Chronic Condition Only							1.81	0.88–3.69	0.11	
Mental Health Condition Only							4.25	2.15–8.37	< 0.001	
Has Both Chronic and Mental Health Condition							6.35	3.13–12.87	< 0.001	
**Survey Type (Community Organization ref.)**							1.26	0.68–2.34		
Constant	0.02	0.00–0.11	< 0.001	0.02	0.00–0.08	< 0.001	0.00	0.00–0.02	< 0.001	

Note. CRBS = Coronavirus Racial Bias Scale, GED = General Education Degree

**Table 4:** Weighted multivariable logistic regression of alcohol use to cope with COVID-19 stressors on stress and impact of discrimination, the Asian American and Native Hawaiian/Pacific Islander COVID-19 Needs Assessment Project, *n* = 3,159

Variables	Model 1			Model 2			Model 3		
	OR	95% CI	*P* - value	OR	95% CI	*P* - value	OR	95% CI	*P* - value
**Exposure and Impact of Discrimination and other non-discrimination-based stressors**									
Racial/ethnic discrimination was greatest source of stress during the COVID-19 Pandemic (No ref.)	1.69	1.19–2.40	0.003	1.30	0.92–1.84	0.14	1.28	0.86–1.89	0.22
Racial/ethnic discrimination impacted family (No ref.)	0.97	0.69–1.37	0.86	0.88	0.62–1.25	0.47	0.87	0.60–1.27	0.48
Coronavirus Racial Bias Scale Mean Score	1.38	1.07–1.78	0.01	1.27	0.98–1.65	0.07	1.14	0.83–1.58	0.42
Sum of COVID Stressors, Except Discrimination				1.18	1.12–1.25	< 0.001	1.17	1.10–1.24	< 0.001
**Demographic Factors**									
Age Category (65 years old ref.)									
18–24 years old							1.57	0.54–4.57	0.40
25–44 years old							3.00	1.08–8.31	0.04
45–64 years old							1.92	0.67–5.48	0.23
Gender (Man ref.)									
Woman							0.60	0.44–0.82	0.002
Non-Binary, Trans, or Other							0.19	0.05–0.74	0.02
Race and Ethnicity (Multiracial Asian ref.)									
Chinese							0.58	0.33–1.01	0.05
Filipino							0.93	0.56–1.55	0.76
Indian							0.83	0.44–1.57	0.56
Vietnamese							0.55	0.31–0.97	0.03
Korean							0.99	0.58–1.71	0.98
Japanese							1.67	0.67–4.17	0.27
Pakistani							0.40	0.11–1.47	0.17
Other Asian Ethnicity							0.29	0.12–0.70	0.006
Multiple Asian Ethnicities							1.01	0.55–1.86	0.98
Marital Status (Married or Living with Partner ref.)									
Single							1.00	0.70–1.42	0.99
Divorced, Separated, or Widowed							0.97	0.47–1.97	0.92
**Acculturative Factors**									
Immigrant to U.S. (Non-immigrant ref.)							0.52	0.37–0.73	< 0.001
Used English in Interview (Other Language ref.)							1.56	0.95–2.54	0.08
**Socioeconomic Factors**									
Educational Attainment (Less than High School ref.)									
High school graduate/GED							0.92	0.35–2.46	0.87
Some college or associate degree							1.03	0.41–2.56	0.96
Bachelor’s degree							1.55	0.63–3.82	0.34
Graduate degree							1.09	0.43–2.76	0.86
Household Income (< $25,000 ref.)									
$25,000 to $34,999							1.17	0.61–2.21	0.64
$35,000 to $49,999							0.71	0.39–1.29	0.26
$50,000 to $74,999							0.89	0.53–1.48	0.65
$75,000 to $99,999							0.75	0.44–1.28	0.29
$100,000 or more							1.18	0.75–1.86	0.48
**Health Factors**									
Preexisting/Current Health Conditions (No Chronic or Mental Conditions ref.)									
Chronic Condition Only							1.23	0.77–1.96	0.38
Mental Health Condition Only							1.85	1.27–2.70	0.001
Has Both Chronic and Mental Health Condition							1.52	0.88–2.64	0.13
**Survey Type (Community Organization ref.)**							0.88	0.60–1.29	0.52
Constant	0.04	0.02–0.10	< 0.001	0.03	0.01–0.07	< 0.001	0.02	0.01–0.11	< 0.001

Note. CRBS = Coronavirus Racial Bias Scale, GED = General Educational Development, equivalent to a high school diploma

## Material and method

### Sample

Data were derived from the Asian subsample of the AA & NH/PI COVID-19 Needs Assessment Project conducted by the Asian American Psychological Association. AA and NH/PI adults 18 years and older were recruited to participate in a two-pronged sampling design through a coalition of community organizations (68% of participants), and an online Qualtrics panel (32% of participants). Community organization recruitment targeted Chinese, Filipino, Korean, Vietnamese, and South Asian ethnicities; but individuals from any Asian ancestry were included. Further information on recruitment of participants is available elsewhere [31–34].

**Table 5:** Weighted multivariable logistic regression of Marijuana/CBD use to cope with COVID-19 stressors on stress and impact of discrimination, the Asian American and Native Hawaiian/Pacific Islander COVID-19 Needs Assessment Project, *n* = 3,159

Variables	Model 1			Model 2			Model 3		
	OR	95% CI	*P* - value	OR	95% CI	*P* - value	OR	95% CI	*P* - value
**Exposure and Impact of Discrimination and other non-discrimination-based stressors**									
Racial/ethnic discrimination was greatest source of stress during the COVID-19 Pandemic (No ref.)	1.34	0.78–2.30	0.28	1.03	0.59–1.82	0.91	1.09	0.61–1.93	0.77
Racial/ethnic discrimination impacted family (No ref.)	1.10	0.65–1.84	0.73	1.00	0.58–1.73	0.99	0.98	0.57–1.69	0.95
Coronavirus Racial Bias Scale Mean Score	2.38	1.62–3.50	< 0.001	2.25	1.51–3.35	< 0.001	1.67	1.06–2.61	0.03
Sum of COVID Stressors, Except Discrimination				1.16	1.08–1.25	< 0.001	1.08	1.00–1.17	0.046
**Demographic Factors**									
Age Category (65 years old ref.)									
18–24 years old							2.86	0.65–12.56	0.16
25–44 years old							3.15	0.75–13.19	0.12
45–64 years old							0.90	0.20–4.03	0.90
Gender (Man ref.)									
Woman							0.85	0.53–1.36	,49
Non-Binary, Trans, or Other							1.62	0.46–5.72	0.45
Race and Ethnicity (Multiracial Asian ref.)									
Chinese							0.28	0.13–0.59	0.001
Filipino							1.44	0.79–2.65	0.23
Indian							0.43	0.17–1.09	0.07
Vietnamese							0.71	0.33–1.53	0.38
Korean							0.71	0.36–1.40	0.32
Japanese							0.19	0.02–1.81	0.15
Pakistani							1.08	0.32–3.70	0.90
Other Asian Ethnicity							0.71	0.22–2.24	0.56
Multiple Asian Ethnicities							1.03	0.50–2.12	0.93
Marital Status (Married or Living with Partner ref.)									
Single							1.16	0.64–2.12	0.63
Divorced, Separated, or Widowed							1.55	0.49–4.95	0.46
**Acculturative Factors**									
Immigrant to U.S. (Non-immigrant ref.)							0.30	0.17–0.53	< 0.001
Used English in Interview (Other Language ref.)							3.80	1.01–14.28	0.048
**Socioeconomic Factors**									
Educational Attainment (Less than High School ref.)									
High school graduate/GED							0.54	0.15–1.93	0.34
Some college or associate degree							0.42	0.13–1.36	0.15
Bachelor’s degree							0.93	0.31–2.77	0.89
Graduate degree							0.62	0.19–2.05	0.43
Household Income (< $25,000 ref.)									
$25,000 to $34,999							0.77	0.33–1.79	0.54
$35,000 to $49,999							0.91	0.42–2.00	0.82
$50,000 to $74,999							0.53	0.26–1.09	0.08
$75,000 to $99,999							0.54	0.25–1.19	0.13
$100,000 or more							0.34	0.17–0.65	0.001
**Health Factors**									
Preexisting/Current Health Conditions (No Chronic or Mental Conditions ref.)									
Chronic Condition Only							1.19	0.63–2.22	0.59
Mental Health Condition Only							2.62	1.61–4.25	> 0.001
Has Both Chronic and Mental Health Condition							2.27	1.03–5.01	0.04
**Survey Type (Community Organization ref.)**							0.54	0.31–0.91	0.02
Constant	0.00	0.00–0.01	< 0.001	0.00	0.00–0.00	< 0.001	0.00	0.00–0.08	0.001

Note. CBD = Cannabidiol, CRBS = Coronavirus Racial Bias Scale, GED = General Educational Development, equivalent to a high school diploma

**Table 6:** Weighted Ordinal Logistic regression of the number of substances used to cope with COVID-19 stressors on stress and impact of discrimination, the Asian American and Native Hawaiian/Pacific Islander COVID-19 Needs Assessment Project, *n* = 3,159

Variables	Model 1			Model 2			Model 3		
	OR	95% CI	*P* - value	OR	95% CI	*P* - value	OR	95% CI	*P* - value
**Exposure and Impact of Discrimination**									
Racial/ethnic discrimination was greatest source of stress during the COVID-19 Pandemic (No ref.)	1.46	1.05–2.03	0.03	1.12	0.80–1.56	0.51	1.26	0.88–1.82	0.21
Racial/ethnic discrimination impacted family (No ref.)	1.10	0.80–1.51	0.56	1.02	0.74–1.42	0.90	0.98	0.69–1.39	0.92
Mean COVID Racial Bias Scale Score	1.50	1.19–1.90	0.001	1.38	1.09–1.75	0.008	1.24	0.92–1.67	0.17
Sum of COVID Stressors, Except Discrimination				1.18	1.13–1.24	< 0.001	1.15	1.08–1.21	< 0.001
**Demographic Factors**									
Age Category (65 years old ref.)									
18–24 years old							1.43	0.57–3.59	0.45
25–44 years old							3.21	1.32–7.81	0.01
45–64 years old							1.79	0.72–4.46	0.21
Gender (Man ref.)									
Woman							0.60	0.46–0.79	< 0.001
Non-Binary, Trans, or Other							0.37	0.12–1.17	0.09
Race and Ethnicity (Multiracial Asian ref.)									
Chinese							0.53	0.32–0.86	0.01
Filipino							1.05	0.67–1.64	0.84
Indian							0.86	0.49–1.50	0.59
Vietnamese							0.62	0.36–1.06	0.08
Korean							1.06	0.65–1.73	0.81
Japanese							1.72	0.77–3.82	0.19
Pakistani							1.01	0.44–2.32	0.99
Other Asian Ethnicity							0.32	0.15–0.71	0.005
Multiple Asian Ethnicities							1.02	0.59–1.77	0.94
Marital Status (Married or Living with Partner ref.)									
Single							1.01	0.73–1.40	0.95
Divorced, Separated, or Widowed							1.29	0.67–2.47	0.45
**Acculturative Factors**									
Immigrant to U.S. (Non-immigrant ref.)							0.46	0.34–0.62	< 0.001
Used English in Interview (Other Language ref.)							1.31	0.83–2.05	0.24
**Socioeconomic Factors**									
Educational Attainment (Less than High School ref.)									
High school graduate/GED							1.35	0.60–3.05	0.47
Some college or associate degree							1.24	0.60–2.56	0.56
Bachelor’s degree							1.43	0.69–2.95	0.33
Graduate degree							1.02	0.49–2.12	0.96
Household Income (< $25,000 ref.)									
$25,000 to $34,999							1.22	0.70–2.16	0.48
$35,000 to $49,999							0.82	0.50–1.36	0.45
$50,000 to $74,999							0.88	0.55–1.41	0.60
$75,000 to $99,999							0.75	0.46–1.22	0.24
$100,000 or more							1.09	0.70–1.71	0.70
**Health Factors**									
Preexisting/Current Health Conditions (No Chronic or Mental Conditions ref.)									
Chronic Condition Only							1.43	0.97–2.11	0.07
Mental Health Condition Only							2.34	1.66–3.30	< 0.001
Has Both Chronic and Mental Health Condition							2.05	1.26–3.34	0.004
**Survey Type (Community Organization ref.)**							0.88	0.63–1.24	0.47

Note. CBD = Cannabidiol, CRBS = Coronavirus Racial Bias Scale, GED = General Educational Development, equivalent to a high school diploma

The survey was offered in English, Bangla, Chinese (traditional and simplified), Hindi, Khmer, Korean, Tagalog, Urdu, and Vietnamese in online, paper, and phone administration formats. Data collection occurred from January 18, 2021 to April 9, 2021. The full survey is included in Appendix A.

For this study, 3,508 respondents who self-identified as Asian for their race, including multiracial individuals, completed the survey; 3,159 individuals were included in the analysis (90.1% complete data). We restrict this analysis to the Asian subsample given that Asian people were the primary targets of discrimination during the COVID-19 pandemic. Most of the missingness was related to age (5.62%), immigration status (2.82%), and income (1.77%). There were significant statistical differences in the ethnic distribution and English language use by completeness (Supplemental Table 1). However, given the high rate of completion, we proceeded with a complete case analysis. NH/PI population analyses are available elsewhere [34].

### Variables

Our three AOD outcomes of interest were assessed by asking participants to indicate “What have you done to cope with your stress related to the COVID-19 outbreak?” by selecting one or more of 12 listed behaviors, which included: (1) using tobacco (e.g., smoking, vaping); (2) drinking alcohol; and (3) using marijuana (e.g., vaping, smoking, eating) or cannabidiol (CBD), henceforth cannabis.

We examined three variables related to COVID-19 discrimination. First, we included whether participants reported if discrimination related to race/ethnicity was among the greatest sources of stress from the COVID-19 pandemic (0 = No, 1 = Yes). Second, we included participants’ reports about whether facing discrimination during COVID-19 had impacted their families (0 = No, 1 = Yes). Finally, we used a five-item scale adapted from Coronavirus Racial Bias Scale (CRBS) [35], to assess perceived racial/ethnic bias due to COVID-19. The study’s five-item scale adapted the wording of the original nine CRBS items to be in a question format rather than a statement format. The five items included were: (1) “Has the U.S. become more physically dangerous for people in your racial/ethnic group because of fear of COVID-19?”; (2) “Because of COVID-19, how likely are people of your race/ethnicity to lose their jobs?”; (3) “How often do you worry about people thinking you have COVID-19 simply because of your race/ethnicity?”; (4) “Due to COVID-19, how often have you been cyberbullied (hate messages/comments directed at you) because of your race/ethnicity?”; and (5) “How much does what politicians say about COVID-19 create bias against people of your racial/ethnic group?” Participants responded to items on a 5-point scale, where a higher score indicates more negative impact or racial bias (e.g., 1 = *Much less dangerous*; 5 = *Much more dangerous* for physical danger). We used a mean score of five items from the modified scale, dropping four items due to poor model fit, and confirming a one-factor structure through confirmatory factor analysis reported elsewhere [32].

We included five domains of covariates that could confound the association between discrimination and drug and alcohol use to cope with COVID-19 stressors. The domains were: COVID-19 stressors, sociodemographic variables, pre-existing and concurrent physical and mental health conditions, and survey medium.

The number of COVID-19 stressors was a sum of 16 possible stressors: physical health concerns, mental health concerns, finances, housing, transportation, caregiving for children or family members, impacts on work, impacts on children, impacts on community, impacts on family members, access to food, access to baby supplies, access to clean water, access to personal care or housing supplies, access to medical care, and concerns over social distancing or quarantine.

Our sociodemographic covariates included age category, gender (man, woman, non-binary or other), ethnicity, and marital status. Race and ethnicity were coded as the following: Multiracial Asian, Chinese, Filipino, Indian, Vietnamese, Korean, Japanese, Pakistani, Other Asian Ethnicity, and Multiethnic Asian. We included two measures of acculturation: whether participants were an immigrant to the U.S. and whether participants completed the survey in English. Socioeconomic covariates included educational attainment and annual household income.

Health covariates included whether participants had a pre-existing or current physical and/or mental health condition: 0 = No chronic or mental health condition, 1 = Chronic condition only, 2 = Mental health condition only, 3 = Has both chronic and mental health condition.

### Analysis plan

Sample weights, as inverse probability weights of sample inclusion, were developed to match the Asian population estimates from the 2019 American Community Survey (ACS) 1-Year estimates from the U.S. Census. Data weights were created based on Asian ethnicity, nativity (foreign born vs. U.S. born), education, household income, gender identity, and age.

We first examined the weighted univariate distribution of the AOD use, discrimination, and associated covariates. Next, we used multivariable binary logistic regression to evaluate the association between smoking, cannabis, and alcohol use to cope with COVID-19 on discrimination. Three models were evaluated per drug and alcohol coping behavior. Model 1 examined the independent associations of racial/ethnic discrimination as a source of stress, whether racial/ethnic discrimination impacted participants’ families, and the mean CRBS score ceteris paribus. We confirmed that multicollinearity among the measures of racial discrimination was not a concern by creating correlation matrices and calculating the variance inflation factor (VIF) for each variable. Our analyses revealed each VIF was below the typical cutoff of VIF = 10 [36]. Model 2 built upon Model 1 and included the number of COVID-19 stressors as an alternative explanation behind AOD use to cope during the COVID-19 pandemic. Model 3 additionally adjusted for demographic, acculturative, socioeconomic, health, and survey type covariates as potential confounders. All analyses were completed using Stata Version 17.0 [37]. A *p* <.05 was used to determine statistical significance for all analyses.

### Sensitivity analyses

We also examined the combination of substances used and number of substances (Range: 0–3). We report the frequencies of the combination of substances used because of low sample sizes across all the possible combinations. However, we used ordinal logistic regression to examine the odds that individuals would use more substances than less substances.

We used the “contrast” command in Stata to examine how each ethnic group’s mean log odds of using each substance and number of substances differed from the grand mean log odds of the entire sample. Given the heterogeneity of experiences for each Asian ethnic group, this analysis allows us to see how each group deviates from the average.

Finally, we examined how the association between discrimination and alcohol and drug use to cope with COVID-19 stressors could be modified by race and ethnicity by including interaction terms of race and ethnicity with each of the three measures of discrimination.

### Ethical considerations

Ethics approval was received from the Association of Asian Pacific Community Health Organizations (AAPCHO) Institutional Review Board and informed consent was obtained from all participants at the beginning of the survey. Additionally, this study did not involve experiments on humans or the use of human tissue samples and was completed in compliance with the Declaration of Helsinki.

## Results

### Survey demographics

Most participants were 25–44 years old (43.1%) with a near even distribution in gender (Table 1). Three largest Asian ethnic groups were Asian Indian (19.0%), Chinese (18.6%), and multiracial Asian (16.8%). Nearly two-thirds of the sample stated that they were immigrants to the U.S. Participants were also highly educated and of higher income. About 48.9% of participants reported having neither a pre-existing or current chronic or mental health condition.

Reported drug and alcohol coping behaviors related to COVID-19 stress were 13% alcohol 4.3% tobacco, and 4.1% cannabis. Most AAs reported using zero substances total to cope with COVID-19 stressors (83.4%), while 12.5% reported using one of the three substances to cope with COVID-19 stressors, and 3.5% reported using two of the three substances to cope with COVID-19 stressors.

Nearly one-fourth (24.5%) of the sample indicated that racial/ethnic discrimination was one of the greatest sources of stress due to the COVID-19 pandemic and 23.5% reported facing discrimination (to the extent that has impacted families). The average CRBS score was 3.57 (SE = 0.02), indicating that AAs endorsed COVID-related racial bias. Finally, independent of discrimination experiences, participants reported experiencing around 3 to 4 stressors related to COVID-19 on average.

### Association of discrimination and tobacco use to cope with COVID-19 stressors

Table 2 presents the results of the weighted multivariable binary logistic regression of tobacco use to cope with COVID-19 stressors on discrimination and the number of COVID-19 stressors. When examining the independent contributions of exposures and impacts of discrimination alone (Model 1), those who reported facing discrimination had higher odds of reporting using tobacco to cope with COVID-19 stressors (OR = 1.85, 95% CI = 1.02, 3.36) compared to those who did not report facing discrimination. Other discrimination measures, CRBS mean score or self-reported racial/ethnic discrimination as greatest source of stress did not have a statistically significant association with tobacco coping use.

The association between facing discrimination and tobacco use became non-significant with the inclusion of the number of COVID-19 stressors (Model 2). Greater reports of COVID-19 stressors were significantly associated with higher odds of tobacco coping use (OR = 1.17, 95% CI = 0.08, 1.26). Of the discrimination factors, facing discrimination was associated with higher odds of tobacco coping use; however, this association was not statistically significant. In the fully adjusted model (Table 2, Model 3), these results remained similar but attenuated. The independent effect of the number of COVID-19 stressors remained significantly associated with tobacco coping use (OR = 1.12, 95% CI = 1.01, 1.25). Each of the three discrimination factors was associated with higher odds of tobacco coping use; however, none of the three factors were statistically significant.

### Association of discrimination with alcohol use to cope with COVID-19 stressors

Table 3 presents the weighted multivariable binary logistic regression for alcohol use to cope. Results were similar to tobacco use. In Model 1, reporting stress related to racial/ethnic discrimination and higher mean CRBS score were significantly associated with higher odds of alcohol coping use. However, when accounting for the number of COVID-19 stressors (Model 2), these associations were attenuated and no longer statistically significant. The number of COVID-19 stressors, however, was associated with higher odds of alcohol coping use (OR = 1.18, 95% CI = 1.12, 1.25). Results remained the same in the fully adjusted model.

### Association of discrimination with cannabis use to cope with COVID-19 stressors

Finally, Table 4 presents the results of cannabis use to cope with COVID-19 stressors. Only mean CRBS score was significantly associated with cannabis coping use (Model 1 OR = 2.38, 95% CI = 1.62, 3.50). This association remained robust when accounting for the number of COVID-19 stressors and after adjusting for demographic, acculturative, socioeconomic, and health factors (Model 3 OR = 1.67, 95% CI = 1.06, 2.61). Finally, the number of COVID-19 stressors remained significantly associated with cannabis coping, like previous analyses.

### Association of discrimination with number of substances use to cope with COVID-19 stressors

We also examined the association between discrimination and pandemic stressors and the number of substances used to cope with weighted ordinal logistic regression (Table 5). In our crude model (Model 1), experiencing stress related to racial/ethnic discrimination and mean CRBS score were significantly associated with greater odds of using more substances. However, when accounting for all COVID-19 stressors (Model 2), only mean CRBS score remained significantly associated with higher odds of using more substances. The sum of COVID-19 stressors was also significantly associated with greater odds of using more substances. Finally, in the fully adjusted model (Model 3), only the sum of COVID-19 stressors was significantly associated with greater odds of using more substances to cope.

### Supplemental analyses

Supplemental analyses (Supplemental Tables 4–8) revealed that most groups have similar log odds of using tobacco to cope with COVID-19 stressors to the sample mean, except for Chinese who had lower log odds (Average Difference (Δ) = -0.57, 95% CI = -1.11, -0.03), and Japanese (Δ = 1.16, 95% CI = 0.08, 2.26) and Pakistani (Δ = 1.42, 95% CI = 0.34, 2.50) people who had higher log odds (See Supplemental Table 4).

Results for alcohol and cannabis were similar to tobacco; most groups have similar odds compared sample mean with some ethnic differences. For alcohol, Japanese (Δ = 0.83, 95% CI = 0.02, 1.65) people had higher log odds while “other Asian” (Δ = -0.93, 95% CI = -1.68, -0.18) had lower log odds compared to the sample mean. For cannabis, multiracial Asian (Δ = 0.48, 95% CI = 0.06, 0.91) and Filipino (Δ = 0.85, 95% CI = 0.39, 1.32) people had higher log odds while Chinese (Δ = -0.79, 95% CI = -1.33, -0.25) people had lower log odds compared to the sample mean.

Finally, we found that the moderation of race/ethnicity on the association of discrimination with substance use was statistically significant for alcohol and cannabis use. However, estimates were unstable.

## Discussion

The current study illustrates that discrimination, COVID-related racial bias, and other stressors impacted AAs during the COVID-19 pandemic. Most participants used alcohol to cope with COVID-19 stressors, followed by tobacco, then cannabis. Whereas stressors related to discrimination based on race/ethnicity and racial bias were associated with some of the outcomes of AOD use to cope with COVID-19 stressors in the discrete and partially adjusted models, only perceived racial bias was associated with cannabis use to cope. Furthermore, the total sum of non-discrimination COVID-19 related stressors was associated with all three AOD outcomes. These findings suggest that stress related to discrimination based on race/ethnicity is associated, to an extent, with use of AOD to cope during the pandemic.

Many AAs in the study reported experiencing racial/ethnic discrimination and bias. This finding is consistent with the experiences of AAs in the U.S. during the pandemic where individuals reported incidents of verbal harassment and physical attacks [1, 38]. For AAs, these stressors are compounding. AAs may also be experiencing economic stress, such as threat to employment security and business [39]. Within the framework of syndemic theory [21], the continued presence of COVID-19 alongside stressors magnified by the pandemic can have health implications such as the use of AOD as a form of coping mechanism within the AA population.

Akin to the general population [9], 16% of the AAs in this study reported using AOD to cope with stress related to the pandemic. While national data show that AAs have lower prevalence of AOD compared to other racial groups [40, 41], previous studies found that there are differences in AOD use by Asian ethnic groups [7, 42]. For instance, national data found that younger AAs compared to older AAs and U.S. born Filipino, Indian Americans, and Koreans were at high-risk for binge drinking and alcohol use disorder compared to other U.S. born Asian ethnic groups [43].

Experiences of discrimination related to race/ethnicity were associated with AOD to cope with COVID-19 stressors to a certain degree but other stressors during the pandemic may be driving the use of AOD to cope with COVID-19 stressors in the AA population. Whereas the stress engendered by discrimination based on race/ethnicity and racial bias have a certain effect on use of AOD to cope, only experience of racial bias related to the COVID-19 pandemic remained consistently associated with marijuana/CBD use when other non-discrimination COVID stressors were considered. Alternatively, the number of non-discrimination COVID-related stressors was consistently associated with all AOD substances. AAs who experience racial bias as one of the stressors during the pandemic might have a preference to use marijuana/CBD when compared to alcohol or tobacco as a coping mechanism. Although studies found evidence on the use of marijuana to cope with anxiety [44], research exploring stressors because of racial bias is understudied and future studies warrant how stress specific to racism is associated with using marijuana/CBD to cope. Other stressors not related to discrimination are influencing participants to use AOD underscoring that AOD remain to be a mechanism of managing stress. While almost a quarter of AAs reported discrimination-related stress and racial bias, perhaps these may not be the primary stressors that are encouraging them to use AOD. For instance, familism is an important cultural trait in AA communities [45], providing financial support for one’s family and keeping family safe from the virus can be stress inducing.

These findings are balanced by a few limitations. First, only alcohol, tobacco, and cannabis use were examined. We did not ask about other substances, such as opioids, which is currently a national crisis [46]. Furthermore, we were unable to fully examine polysubstance use due to low sample sizes. In our weighted sample, only 4.1% of participants used more than 2 substances.

Second, we only examined the perceived mechanism underlying substance use behavior in coping with COVID-19 stressors rather than general substance use behavior. It is possible that some individuals may use AOD habitually or recreationally and not as a coping mechanism to deal with COVID-19 related stress. National studies have found that alcohol use increased by 5% among adults ages 21 to 24 years old while cannabis use has increased by 1.2% among adults 25 years and older between 2018 and 2020 [10]. While these trends may be reflective of potential use to cope with COVID-19 stress, it was unclear if these trends are confounded by increase recreational use due to destigmatizing of cannabis.

Third, we were unable to fully disaggregate the type of cannabis use to cope with COVID-19 stressors. Different cannabis types (e.g., CBD, marijuana) have different safety and psychoactive profiles that may perpetuate single versus chronic use. Future studies should consider disentangling cannabis as well as other substances to examine the specific type used.

Fourth, our measures of discrimination did not ask about the timing or frequency of discrimination that AAs may have faced. Instead, it asked if racial/ethnic discrimination was a major source of stress from the pandemic. We also asked if facing discrimination impacted participants’ families. Thus, participants may have vicariously experienced discrimination despite never experiencing any interpersonal incidents. Studies have noted how greater reports of vicarious racism and vigilance among Black and AA people were associated with greater depression, anxiety [22] and worse sleep [47].

Finally, although this study was a national survey of AAs, it should be acknowledged that we were not able to account for the full breadth of AA ethnic groups. While examining differences in AOD use by Asian ethnic group was not the main focus of this study, we do provide some preliminary comparisons to the mean level of substance use. In addition, there may be other factors at the state and local level that could affect the association between discrimination and substance use. For example, living in a state or community with fewer Asian individuals may expose Asian people to more severe experiences of discrimination.

## Conclusion

About one in six AAs in the study reported using alcohol, tobacco and/or cannabis in coping with COVID-19 pandemic-related stress. Experiences of racially related discrimination in the form of racial bias was associated with cannabis use but not for tobacco or alcohol use. Instead, other non-discrimination stressors experienced during the pandemic were a consistent correlate of AAs’ AOD use to cope with stress. Policymakers, researchers, and community organizers should conduct studies to understand how COVID-19 related stressors are impacting different groups within the AA population, specific to the use of different AOD types, and implement policies that address racism targeting AAs and resources addressing AOD use.

## Electronic supplementary material

Below is the link to the electronic supplementary material.

Supplementary Material 1

Supplementary Material 2

## Data Availability

Data and code will be provided upon reasonable request by emailing the corresponding author, janice.tsoh@ucsf.edu.

## Abbreviations

AA: Asian Americans
AAPCHO: Association of Asian Pacific Community Health Organizations
AAPI: Asian American and Pacific Islander
ACS: American Community Survey
AOD: Alcohol and other drugs
CBD: Cannabidiol
CI: Confidence Interval
CRBS: Coronavirus Racial Bias Scale
NH: Non-Hispanic
NH/PI: Native Hawaiian or Pacific Islander
OR: Odds Ratio
U.S.: United States
VIF: Variance Inflation Factor
