# Supplementary material for: Perceived discrimination and coping with substance use among Asian Americans during the COVID-19 pandemic: a cross-sectional analysis
Source: BMC Public Health. 2025 Feb 20;25:698. doi: 10.1186/s12889-025-21824-2 (PMC11841164; doi:10.1186/s12889-025-21824-2)
Supplement: Supplementary file 1 — Supplementary Material 1 [file 12889_2025_21824_MOESM1_ESM.docx]

Supplemental Table 1. Demographic Characteristics by Missingness

|  | Missing  (n = 349) | In Sample  (n = 3,159) | *P-value* |
| --- | --- | --- | --- |
|  | % (n) | % (n) |  |
| Asian Ethnicity |  |  | .003 |
| Multiracial Asian | 7.16 (24) | 7.82 (247) |  |
| Chinese | 25.79 (90) | 22.13 (699) |  |
| Filipino | 10.89 (38) | 17.82 (563) |  |
| Indian | 12.03 (42) | 9.43 (298) |  |
| Vietnamese | 10.60 (37) | 13.52 (437) |  |
| Korean | 16.91 (59) | 13.01 (411) |  |
| Japanese | 2.01 (7) | 1.68 (53) |  |
| Pakistani | 3.72 (13) | 2.18 (69) |  |
| Other Asian | 5.73 (20) | 8.04 (254) |  |
| Multiethnic Asian | 5.73 (20) | 8.04 (254) |  |
| English Used In Interview | 71.35 (249) | 87.75 (2,772) | < .001 |

Supplemental Table 2. Bivariate Distribution of Stress Related to Racial/Ethnic Discrimination and Impact of Discrimination on Families by Tobacco, Alcohol, and Marijuana/CBD Use to Cope with Stressors

|  | Tobacco Use | | | Alcohol Use | | | Marijuana/CBD Use | | |
| --- | --- | --- | --- | --- | --- | --- | --- | --- | --- |
|  | Yes | No |  | Yes | No |  | Yes | No |  |
| Unweighted n | 147 | 3012 |  | 455 | 2704 |  | 190 | 2969 |  |
| Racial/ethnic discrimination stress | % (n) | % (n) | p-value | % (n) | % (n) | p-value | % (n) | % (n) | p-value |
| Yes | 30.8 (57) | 24.2 (958) | .18 | 37.7 (214) | 22.5 (801) | < .001 | 43.2 (101) | 23.7 (914) | < .001 |
| No | 69.2 (90) | 75.8 (2054) |  | 62.3 (241) | 77.5 (1903) |  | 56.8 (89) | (76.3 2055) |  |
| Racial/ethnic discrimination impacted family | % (n) | % (n) | p-value | % (n) | % (n) | p-value | % (n) | % (n) | p-value |
| Yes | 36.8 (63) | 23.0 (864) | .006 | 30.6 (186) | 22.5 (723) | .007 | 38.3 (87) | 22.9 (822) | < .001 |
| No | 63.2 (84) | 77.0 (2166) |  | 69.4 (269) | 77.5 (1981) |  | 61.7 (103) | 77.1 (2147) |  |

Note. Percentages represent weighted column percentages. N’s represent unweighted n.

Supplemental Table 3. Correlation Matrix of Discrimination, COVID-19 Stressors, and Drug and Alcohol Use, The Asian American and Native Hawaiian/Pacific Islander COVID-19 Needs Assessment Project (n = 3,159)

|  | Discrimination Stress | Discrimination Impacted Family | CRBS Mean | Total # of Stressors (No Discrimination) | Tobacco Use | Alcohol Use | Marijuana/CBD Use |
| --- | --- | --- | --- | --- | --- | --- | --- |
| Discrimination Stress | 1 |  |  |  |  |  |  |
| Discrimination Impacted Family | 0.47*** | 1 |  |  |  |  |  |
| CRBS Mean | 0.47*** | 0.40*** | 1 |  |  |  |  |
| Total # of Stressors (No Discrimination) | 0.38*** | 0.26*** | 0.32*** | 1 |  |  |  |
| Tobacco Use | 0.03 | 0.07*** | 0.04 | 0.11*** | 1 |  |  |
| Alcohol Use | 0.13*** | 0.11*** | 0.14*** | 0.20*** | 0.24*** | 1 |  |
| Marijuana/CBD Use | 0.11*** | 0.10*** | 0.14*** | 0.15*** | 0.20*** | 0.29*** | 1 |

Note. CRBS = Coronavirus Racial Bias Scale, CBD = Cannabidiol, * p < .05, ** p < .01, *** p < .001.

Supplemental Table 4. Asian Ethnic Group Differences from the Mean Log Odds of Tobacco Use to Cope

| Asian Ethnic Category | Average Difference in Log Odds from Overall Mean Log Odds | *P-value* | 95% CI |
| --- | --- | --- | --- |
| Multiracial Asian | -0.07 | .81 | -0.62, 0.48 |
| Chinese | -0.57 | .04 | -1.11, -0.03 |
| Filipino | 0.05 | .83 | -0.43, 0.54 |
| Indian | 0.51 | .10 | -0.09, 1.12 |
| Vietnamese | -0.28 | .42 | -0.98, 0.41 |
| Korean | 0.49 | .12 | -0.13, 1.12 |
| Japanese | 1.16 | .034 | 0.08, 2.26 |
| Pakistani | 1.42 | .01 | 0.34, 2.50 |
| Other Asian | -0.52 | .34 | -1.58, 0.54 |
| Multiethnic Asian | -0.57 | .30 | -1.66, 0.51 |

Note. Models account for stress and impact of discrimination, Coronavirus Racial Bias Scale mean score, other COVID-19 stressors, demographic, immigration, socioeconomic and health factors (Model 3 of Table 3).

Supplemental Table 5. Asian Ethnic Group Differences from the Mean Log Odds of Alcohol Use to Cope

| Asian Ethnic Category | Average Difference in Log Odds from Overall Mean Log Odds | *P-value* | 95% CI |
| --- | --- | --- | --- |
| Multiracial Asian | 0.32 | .10 | -0.05, 0.69 |
| Chinese | -0.23 | .17 | -0.55, 0.10 |
| Filipino | 0.25 | .10 | -0.05, 0.54 |
| Indian | 0.13 | .57 | -0.32, 0.58 |
| Vietnamese | -0.28 | .17 | -0.68, 0.12 |
| Korean | 0.31 | .10 | -0.05, 0.68 |
| Japanese | 0.83 | .045 | 0.02, 1.65 |
| Pakistani | -0.59 | .34 | -1.81, 0.62 |
| Other Asian | -0.93 | .02 | -1.68, -0.18 |
| Multiethnic Asian | 0.33 | .18 | -0.15, 0.80 |

Note. Models account for stress and impact of discrimination, Coronavirus Racial Bias Scale mean score, other COVID-19 stressors, demographic, immigration, socioeconomic and health factors (Model 3 of Table 4).

Supplemental Table 6. Asian Ethnic Group Differences from the Mean Log Odds of Marijuana/CBD Use to Cope

| Asian Ethnic Category | Average Difference in Log Odds from Overall Mean Log Odds | *P-value* | 95% CI |
| --- | --- | --- | --- |
| Multiracial Asian | 0.48 | .023 | 0.06, 0.91 |
| Chinese | -0.79 | .004 | -1.33, -0.25 |
| Filipino | 0.85 | < .001 | 0.39, 1.32 |
| Indian | -0.37 | .31 | -1.07, 0.34 |
| Vietnamese | 0.14 | .68 | -0.54, 0.83 |
| Korean | 0.14 | .63 | -0.42, 0.70 |
| Japanese | -1.16 | .29 | -3.29, 0.97 |
| Pakistani | 0.57 | .34 | -0.59, 1.72 |
| Other Asian | 0.14 | .79 | -0.87, 1.14 |
| Multiethnic Asian | 0.52 | .11 | -0.11, 1.14 |

Note. Models account for stress and impact of discrimination, Coronavirus Racial Bias Scale mean score, other COVID-19 stressors, demographic, immigration, socioeconomic and health factors (Model 3 of Table 5).

Supplemental Table 7. Asian Ethnic Group Differences from the Mean Log Odds of Number of Substances Used to Cope

| Asian Ethnic Category | Average Difference in Log Odds from Overall Mean Log Odds | p-value | 95% CI |
| --- | --- | --- | --- |
| Multiracial Asian | 0.27 | .101 | -0.05, 0.59 |
| Chinese | -0.37 | .01 | -0.67, -0.08 |
| Filipino | 0.31 | .02 | 0.04, 0.59 |
| Indian | 0.11 | .59 | -0.30, 0.52 |
| Vietnamese | -0.21 | .30 | -0.62, 0.19 |
| Korean | 0.33 | .06 | -0.01, 0.67 |
| Japanese | 0.81 | .02 | 0.10, 1.51 |
| Pakistani | 0.27 | .48 | -0.48, 1.03 |
| Other Asian | -0.86 | .01 | -1.51, -0.21 |
| Multiethnic Asian | 0.29 | .19 | -0.14, 0.72 |

Note. Models account for stress and impact of discrimination, Coronavirus Racial Bias Scale mean score, other COVID-19 stressors, demographic, immigration, socioeconomic and health factors (Model 3 of Table 6).

Supplemental Table 8. Adjusted Wald Test of Significance for Moderation of Race/Ethnicity on the Association of Discrimination on Substance Use

|  | Tobacco | | Alcohol | | Marijuana/CBD | | Number of Substances | |
| --- | --- | --- | --- | --- | --- | --- | --- | --- |
| Interaction Term | *F* | *P-Value* | *F* | *P-Value* | *F* | *P-Value* | *F* | *P-Value* |
| Stress of Discrimination x Race/Ethnicity | 0.86 | .55 | 0.63 | .77 | 1.45 | .17 | 1.19 | .30 |
| Discrimination Family Impact x Race/Ethnicity | 1.54 | .13 | 2.08 | .03 | 2.34 | .02 | 1.14 | .33 |
| CRBS x Race/Ethnicity | 0.94 | .48 | 1.05 | .40 | 35.07 | < .001 | 0.87 | .55 |

Note. “Stress of Discrimination” = Racial/ethnic discrimination was greatest source of stress during the COVID-19 pandemic. “Discrimination Family Impact” = Racial/ethnic discrimination impacted family. CRBS = Coronavirus Racial Bias Scale. All Models adjust for age, gender, race/ethnicity, marital status, immigrant status, educational attainment, annual income, health status, and survey type.
