# Supplementary material for: Perceived discrimination and coping with substance use among Asian Americans during the COVID-19 pandemic: a cross-sectional analysis
Source: BMC Public Health. 2025 Feb 20;25:698. doi: 10.1186/s12889-025-21824-2 (PMC11841164; doi:10.1186/s12889-025-21824-2)
Supplement: Supplementary file 2 — Supplementary Material 2 [file 12889_2025_21824_MOESM2_ESM.pdf]

AAPI COVID-19 Needs Assessment Survey

Q1) What is your race? *Select all that apply.*

- ○ Asian
- ○ Native Hawaiian or Pacific Islander
- ○ Native American or Alaskan Native
- ○ African American or Black
- ○ White, Non-Hispanic/Latino
- ○ Hispanic/Latino
- ○ Middle Eastern or North African

Q2) How important is/are your race(s) to your identity?

- ○ Extremely important
- ○ Very important
- ○ Somewhat important
- ○ Not at all important

Q3) Only answer this question if you selected "**Asian**" in Q1: What term(s) best express how you describe your ethnic identity *Select all that apply.*

- ○ Chinese
- ○ Filipino
- ○ Indian
- ○ Vietnamese
- ○ Korean
- ○ Japanese
- ○ Pakistani
- ○ Cambodian
- ○ Hmong
- ○ Thai
- ○ Laotian
- ○ Bangladeshi
- ○ Burmese
- ○ Indonesian
- ○ Nepalese
- ○ Sri Lankan
- ○ Malaysian
- ○ Bhutanese
- ○ Mongolian
- ○ Okinawan
- ○ Other, please specify: ____

Q4) Only answer this question if you selected "**Native Hawaiian or Pacific Islander**" in Q1: What term(s) best expresses how you describe your ethnic identity? *Select all that apply.*

- ○ Native Hawaiian
- ○ Samoan
- ○ Tongan
- ○ Fijian
- ○ Marshallese
- ○ CHamoru
- ○ Chuukese
- ○ Other, please specify: ____

Q5) How important is your ethnicity to your identity?

- ○ Extremely important
- ○ Very important
- ○ Somewhat important
- ○ Not at all important

Q6) Which of the following do you need more help getting during the COVID-19 pandemic? *Select all that apply.*

- ○ Food
- ○ Housing
- ○ Utilities (electricity, gas, water, sewer, trash collection, phone, cable)
- ○ Internet/Wi-Fi
- ○ Unemployment services
- ○ Health services
- ○ Mental health services
- ○ Emergency services (EMS, Fire, Police)
- ○ Legal assistance
- ○ Childcare
- ○ Faith-based or spiritual care
- ○ Other, please specify: ____
- ○ None of the above

Q7) How has the COVID-19 pandemic impacted your family's life? *Select all that apply.*

- ○ Unable to access healthcare
- ○ We lost work/jobs
- ○ We lost income
- ○ Facing discrimination
- ○ Impacted my social/religious activities
- ○ Disrupted education of me or my family members
- ○ Other, please specify: ____
- ○ None of the above

Now we are going to ask about your employment.

Q8) Have you, or has anyone in your household experienced a loss of employment income **since March 13, 2020**? *Select only one answer.*

- ○ Yes
- ○ No

Q9) Do you expect that you or anyone in your household will experience a loss of employment income in the **next 4 weeks** because of the COVID-19 pandemic? *Select only one answer.*

- ○ Yes
- ○ No

Q10) In the **last 7 days**, did you do **ANY** work for either pay or profit? *Select only one answer.*

- ○ Yes
- ○ No

Q11) Only answer this question if you selected "**Yes**" for Q10: Are you employed by government, by a private company, a nonprofit organization or were you self-employed or working in a family business? *Select only one answer.*

- ○ Government
- ○ Private company
- ○ Non-profit organization including tax exempt and charitable organizations
- ○ Self-employed
- ○ Working in a family business

Q12) Only answer this question if you selected "**No**" for Q10: What is your main reason for not working for pay or profit? *Select only one answer.* I did not work because:

- ○ I did not want to be employed at this time
- ○ I am/was sick with COVID-19 symptoms
- ○ I am/was caring for someone with COVID-19 symptoms
- ○ I am/was caring for children not in school or daycare
- ○ I am/was caring for an elderly person
- ○ I was concerned about getting or spreading COVID-19
- ○ I am/was sick (not COVID-19 related) or disabled
- ○ I am retired
- ○ My employer experienced a reduction in business (including furlough) due to the COVID-19 pandemic
- ○ I am/was laid off due to the COVID-19 pandemic
- ○ My employer closed temporarily due to the COVID-19 pandemic
- ○ My employer went out of business due to the COVID-19 pandemic
- ○ Other; please specify: ____

Q13) Only answer this question if you selected "**No**" for Q10: Are you receiving pay for the time you are not working? *Select only one answer.*

- ○ Yes, I use paid leave
- ○ Yes, I receive full pay but do not have to take leave
- ○ Yes, I receive partial pay
- ○ No, I receive no pay

Q14) Getting enough food can also be a problem for some people. Which of these statements best describes the food eaten in your household before **March 13, 2020**? *Select only one answer.*
- Enough of the kinds of food I (we) wanted to eat
- Enough, but not always the kinds of food I (we) wanted to eat
- Sometimes not enough to eat
- Often not enough to eat

Q15) In the **last 7 days**, which of these statements best describes the food eaten in your household? *Select only one answer.*
- Enough of the kinds of food I (we) wanted to eat
- Enough, but not always the kinds of food I (we) wanted to eat
- Sometimes not enough to eat
- Often not enough to eat

Q16) Only answer this question if you **did not** select option 1 in Q15: Why did you not have enough to eat (or not what you wanted to eat)? *Select all that apply.*
- Couldn't afford to buy more food
- Couldn't get out to buy food (for example, didn't have transportation, or had mobility or health problems that prevented you from getting out)
- Afraid to go or didn't want to go out to buy food
- Couldn't get groceries or meals delivered to me
- The stores didn't have the food I wanted
- None of the above

Q17) How confident are you that your household will be able to afford the kinds of food you need for the **next four weeks**? *Select only one answer.*
- Not at all confident
- Somewhat confident
- Moderately confident
- Very confident

Q18) What is your height?
- In inches: ____
- OR in centimeters: ____
- Don't know
- Decline to state

Q19) What is your weight?
- In pounds: ____
- OR in kilograms: ____
- Don't know
- Decline to state

Q20) Please write what your age was (in years) on **January 1, 2021**: ____________________________

Q21) **Before the COVID-19 pandemic** (before March 13, 2020), would you say your health in general was excellent, very good, good, fair, or poor? *Select only one answer.*
- Excellent
- Very good
- Good
- Fair
- Poor

Q22) **Currently**, would you say your health in general is excellent, very good, good, fair, or poor? *Select only one answer.*

- Excellent
- Very good
- Good
- Fair
- Poor

Q23) Over the **last 7 days**, how often have you been bothered by **feeling nervous, anxious, or on edge**? *Select only one answer.*

- Not at all
- Several days
- More than half the days
- Nearly every day

Q24) Over the **last 7 days**, how often have you been bothered by **not being able to stop or control worrying**? *Select only one answer.*

- Not at all
- Several days
- More than half the days
- Nearly every day

Q25) Over the **last 7 days**, how often have you been bothered by **having little interest or pleasure in doing things**? *Select only one answer.*

- Not at all
- Several days
- More than half the days
- Nearly every day

Q26) Over the **last 7 days**, how often have you been bothered by **feeling down, depressed, or hopeless**? *Select only one answer.*

- Not at all
- Several days
- More than half the days
- Nearly every day

Q27) Have you ever been told by a doctor or nurse that you have: *Select all that apply?*

- High blood pressure (hypertension)
- Diabetes (not including pre-diabetes)
- Cardiovascular disease (including blocked or hardening of the arteries, angina, or chest pain, heart attack, stroke or mini stroke)
- Congestive heart failure
- Lung disease [asthma or COPD (emphysema, chronic bronchitis)]
- Cancer that you are getting treatment for now
- Autoimmune disease (like lupus, rheumatoid arthritis, psoriasis)
- Kidney disease, including weak or failing kidneys (Do NOT include kidney stones or problems with urinating)
- Low immunity (on any medication that decreases your immunity, such as for a transplant or an immune disease)
- Anxiety
- Depression
- Post-traumatic stress disorder (PTSD)
- Other mental health conditions
- None of the above

Q28) How have you changed your food habits since the COVID-19 pandemic? *Select all that apply.*
- ○ Did not change my food habits
- ○ Started using online grocery and food delivery
- ○ Ordering food from restaurants or takeout more
- ○ Ordering food from restaurants or takeout less
- ○ Meal rationing/skipping meals
- ○ Using food pantry or food bank
- ○ Using city or community-based organization food delivery program like Meals on Wheels
- ○ Getting food from my child/children's school
- ○ Getting food from neighbors
- ○ Eating or using expired or spoiling food
- ○ Cooking more at home
- ○ Cooking less at home
- ○ Other; please specify: ____

Q29) Compared to before COVID-19, are you **exercising (being physically active)** less, more or the same?
- ○ A lot less
- ○ Somewhat less
- ○ The same
- ○ Somewhat more
- ○ A lot more

Q30) Compared to before COVID-19, are you **sedentary/sitting** more, less or the same amount as you were before?
- ○ A lot less
- ○ Somewhat less
- ○ The same
- ○ Somewhat more
- ○ A lot more

Q31) What have been your greatest sources of stress from the COVID-19 pandemic? *Select all that apply.*
- ○ Physical health concerns
- ○ Mental health concerns
- ○ Financial concerns
- ○ Housing concerns
- ○ Transportation concerns
- ○ Caregiving responsibilities (for example, caring for children, family members)
- ○ Impact on work
- ○ Impact on your child
- ○ Impact on your community
- ○ Impact on family members
- ○ Access to food
- ○ Access to baby supplies (e.g., formula, diapers, wipes)
- ○ Access to clean water for hand washing etc.
- ○ Access to personal care products or household supplies
- ○ Access to medical care, including mental health care
- ○ Social distancing or being quarantined
- ○ Discrimination due to my race/ethnicity
- ○ I am not stressed about the COVID-19 pandemic
- ○ None of the above

<u>Q32)</u> What have you done to cope with your stress related to the COVID-19 pandemic? *Select all that apply.*

- Talking with friends and family (e.g., by phone, text, or video)
- Talking to my healthcare providers more frequently, including mental healthcare provider (e.g., therapist, psychologist, counselor)
- Meditation and/or mindfulness practices
- Religious or spiritual practices (e.g., praying, reading religious texts)
- Television watching or other "screen time" activities (e.g., video games, social media)
- Hobbies and indoor activities (e.g., painting, playing a musical instrument, reading, cooking/baking)
- Participated in civic engagement activities (e.g., joined/participated in a community organization, registered to vote, organized/advocated for my community, attended a protest, contacted an elected official)
- Exercise (e.g., running, walking, sports, dance)
- Eating, including snacking
- Sleeping, including napping or lying down in bed
- Drinking alcohol
- Using tobacco (e.g., smoking, vaping)
- Using marijuana (e.g., vaping, smoking, eating) or cannabidiol (CBD)
- Other, please specify: ____
- I have not done any of these things to cope with the COVID-19 pandemic

<u>Q33)</u> Since becoming aware of the COVID-19 pandemic, how often have you felt happy and satisfied with your life?

- Not at all
- Rarely
- Sometimes
- Often
- Very Often

<u>Q34)</u> Are you currently covered by any of the following types of health insurance or health coverage plans? *Select all that apply.*

- Insurance through a current or former employer or union (through yourself or another family member)
- Insurance purchased directly from an insurance company, including marketplace coverage (through yourself or another family member)
- Medicare, for people 65 and older, or people with certain disabilities
- Medicaid, Medical Assistance, or any kind of government-assistance plan for those with low incomes or a disability
- TRICARE or other military health care
- VA (including those who have ever used or enrolled for VA health care)
- Indian Health Services
- Other
- I am not currently covered by any health insurance or health coverage plan

<u>Q35)</u> At any time in the **last 4 weeks**, did you DELAY getting medical care <u>because of the COVID-19 pandemic</u>? *Select only one answer.*

- Yes
- No
- I did not have any medical needs in the last 4 weeks

<u>Q36)</u> At any time in the **last 4 weeks**, did you need medical care for <u>something other than COVID-19</u>, but DID NOT GET IT because of the COVID-19 pandemic? *Select only one answer.*

- Yes
- No
- I did not have any medical needs in the last 4 weeks

<u>Q37)</u> In what ways has the COVID-19 pandemic affected your overall healthcare? *Select all that apply.*
- I did not go to healthcare appointments because I was concerned about entering my healthcare provider's office
- My healthcare provider canceled appointments
- My healthcare provider changed to phone or online visits
- My healthcare provider told me to self-isolate or quarantine
- None of these apply

<u>Q38)</u> How difficult was it to **see a healthcare provider** if you or your family needed to **BEFORE** the COVID-19 pandemic?
- Not difficult
- Somewhat difficult
- Very difficult

<u>Q39)</u> How difficult has it been to **see a healthcare provider** if you or your family needed to **DURING** the COVID-19 pandemic?
- Not difficult
- Somewhat difficult
- Very difficult

<u>Q40)</u> How difficult was it to **get routine/essential medications** for you and/or your family **BEFORE** the COVID-19 pandemic?
- Not difficult
- Somewhat difficult
- Very difficult

<u>Q41)</u> How difficult was it to **get routine/essential medications** for you and/or your family **DURING** the COVID-19 pandemic?
- Not difficult
- Somewhat difficult
- Very difficult

<u>Q42)</u> How much has the COVID-19 pandemic changed stress and discord in the family?
- Improved. Family members have become closer with one another.
- None. There has been no change in stress and discord in the family.
- Mild. Family members occasionally short-tempered with one another; no physical violence.
- Moderate. Family members frequently short-tempered with one another; and/or children in the home getting in physical fights with one another.
- Severe. Family members frequently short-tempered with one another and adults in the home throwing things at one another, and/or knocking over furniture, and/or hitting and/or harming one another.

<u>Q43)</u> What were some of the challenges of remote learning for you and/or your child? *Select all that apply.*
- Lack of technological equipment
- Lack of WI-FI access
- Lack of high-speed internet access
- Inability to concentrate/study because all family members are present at home
- Teachers/professors not accommodating of the student's circumstances
- Language barriers in understanding the material
- Other, please describe: ____
- I and/or my child did not experience any challenges with remote learning
- This question does not apply to me (I'm not a student, and I don't have a child who is a student)

Q44) Prior to the COVID-19 pandemic, how supported did you feel by your social network?

- ○ Not supported
- ○ Mostly unsupported
- ○ Somewhat unsupported
- ○ Neutral
- ○ Somewhat supported
- ○ Mostly supported
- ○ Very supported

Q45) Currently (during the COVID-19 pandemic), how supported do you feel by your social network?

- ○ Not supported
- ○ Mostly unsupported
- ○ Somewhat unsupported
- ○ Neutral
- ○ Somewhat supported
- ○ Mostly supported
- ○ Very supported

Q46) Since the breakout of the COVID-19 pandemic, I have received emotional support from family or friends when needed

- ○ Strongly agree
- ○ Agree
- ○ Neither agree nor disagree
- ○ Disagree
- ○ Strongly disagree

Q47) Since the breakout of the COVID-19 pandemic, I have received tangible support (e.g., financial, practical) from family or friends when needed

- ○ Strongly agree
- ○ Agree
- ○ Neither agree nor disagree
- ○ Disagree
- ○ Strongly disagree

Q48) Since the breakout of the COVID-19 pandemic, I am (or have been) there to listen to other's problems when needed

- ○ Strongly agree
- ○ Agree
- ○ Neither agree nor disagree
- ○ Disagree
- ○ Strongly disagree

Q49) Since the breakout of the COVID-19 pandemic, I have helped others with financial or practical support

- ○ Strongly agree
- ○ Agree
- ○ Neither agree nor disagree
- ○ Disagree
- ○ Strongly disagree

Q50) Please indicate the extent to which you view the COVID-19 pandemic as having either a positive or negative impact on your life.

- Extremely negative
- Moderately negative
- Somewhat negative
- No impact
- Slightly positive
- Moderately positive
- Extremely positive

Q51) Since the breakout of the COVID-19 pandemic, I have greater appreciation for my family and close friends.

- Strongly agree
- Agree
- Neither agree nor disagree
- Disagree
- Strongly disagree

Q52) Since the breakout of the COVID-19 pandemic, I have deeper appreciation for life.

- Strongly agree
- Agree
- Neither agree nor disagree
- Disagree
- Strongly disagree

Q53) Since the breakout of the COVID-19 pandemic, I have been more grateful for each day.

- Strongly agree
- Agree
- Neither agree nor disagree
- Disagree
- Strongly disagree

Q54) Since the breakout of the COVID-19 pandemic, I have been more accepting of things I cannot change.

- Strongly agree
- Agree
- Neither agree nor disagree
- Disagree
- Strongly disagree

Q55) Since the breakout of the COVID-19 pandemic, I have found new ways of connecting with family and friends.

- Strongly agree
- Agree
- Neither agree nor disagree
- Disagree
- Strongly disagree

Q56) Which state do you **CURRENTLY** live in?_______________________________________

Please answer the following questions on your beliefs about how COVID-19 is affecting people of your race/ethnicity.

<u>Q57)</u> Has the U.S. become more physically dangerous for people in your racial/ethnic group because of fear of COVID-19?
- ○ Much more dangerous
- ○ Slightly more dangerous
- ○ Not more dangerous
- ○ Slightly less dangerous
- ○ Much less dangerous

<u>Q58)</u> Because of COVID-19, how likely are people of your race/ethnicity to lose their jobs?
- ○ Much more likely
- ○ Slightly more likely
- ○ Not more likely
- ○ Less likely
- ○ Much less likely

<u>Q59)</u> How often do you worry about people thinking you have COVID-19 simply because of your race/ethnicity?
- ○ Constantly
- ○ Very often
- ○ Somewhat often
- ○ Rarely
- ○ Never

<u>Q60)</u> How much do social and mass media reports about COVID-19 change attitudes against people of your racial/ethnic group?
- ○ Much more positive
- ○ Slightly more positive
- ○ No change
- ○ Slightly more negative
- ○ Much more negative

<u>Q61)</u> Compared to other groups, what is the risk of getting COVID-19 for people of your race/ethnicity?
- ○ Much more likely
- ○ Slightly more likely
- ○ Not more likely
- ○ Slightly less likely
- ○ Much less likely

<u>Q62)</u> Compared to other groups, how is the quality of COVID-19 healthcare for people of your race/ethnicity?
- ○ Much better
- ○ Slightly better
- ○ Not better/the same
- ○ Slightly worse
- ○ Much worse

Q63) Due to COVID-19, how often have you been cyberbullied (hate messages/comments directed at you) because of your race/ethnicity?

- o Never
- o One or two times
- o Two or three times a month
- o Once a week
- o Nearly every day

Q64) Since COVID-19, have you seen a change in the amount of cyberbullying of people of your race/ethnicity?

- o Greatly increased
- o Slightly increased
- o No change
- o Slightly decreased
- o Greatly decreased

Q65) How much does what politicians say about COVID-19 create bias against people of your racial/ethnic group?

- o Strongly increase bias
- o Slightly increase bias
- o No effect
- o Slightly decrease bias
- o Strongly decrease bias

Q66) Which of the following election and civic opportunities are you aware of? *Select all that apply.*

- o November 2020 general election
- o 2020 Census
- o None of the above

Q67) Only answer this question if you selected "**November 2020 general election**" for Q66: Did you do any of the following? *Select all that apply.*

- o Received information about November 2020 voter registration deadlines or the election.
- o Registered to vote for the November 2020 Election.
- o Voted by mail in the November 2020 Election.
- o Voted early in the November 2020 Election.
- o Voted on Election Day in the November 2020 Election.
- o I did not vote in the November 2020 Election because I am not eligible to vote.
- o I did not vote in the November 2020 Election because I did not receive my ballot.
- o I did not vote in the November 2020 Election because I did not know who or what to vote for.
- o Other reason you did not vote (please specify): ____

Q68) Only answer this question if you selected "**2020 Census**" for Q66: Did you do any of the following? *Select all that apply.*

- o Received information about the 2020 Census and deadlines
- o Completed the 2020 Census online
- o Completed the 2020 Census over the phone
- o Completed the 2020 Census on paper
- o Completed the 2020 Census by speaking with an enumerator (someone who stopped by your home and interviewed you)

Q69) Is your house or apartment...? *Select only one answer.*
- ○ Owned by you or someone in this household free and clear
- ○ Owned by you or someone in this household with a mortgage or loan (including home equity loans)
- ○ Rented
- ○ Occupied without payment or rent

Q70) Only answer this question if you selected **option 3 or 4** in Q69: Did you pay your last month's rent or mortgage on time? *Select only one answer.*
- ○ Yes
- ○ No
- ○ Payment was deferred

Q71) Only answer this question if you selected **option 3 or 4** in Q69: How confident are you that your household will be able to pay your next rent or mortgage payment on time? *Select only one answer.*
- ○ No confidence
- ○ Slight confidence
- ○ Moderate confidence
- ○ High confidence
- ○ Payment is/will be deferred

Q72) Do you think you have or had COVID-19?
- ○ Definitely yes
- ○ Probably yes
- ○ Unsure
- ○ Probably not
- ○ Definitely not

Q73) Have you been tested for COVID-19?
- ○ No, I never tried to get tested
- ○ No, I tried to get tested but was not able to
- ○ Yes, once
- ○ Yes, 2-3 times
- ○ Yes, more than 3 times

Q74) Did you have any of the following? *Select all that apply.*
- ○ Symptoms of COVID-19
- ○ A positive test for COVID-19
- ○ Close contact with someone who had COVID-19
- ○ A healthcare provider ever told you that you had COVID-19
- ○ None of the above

Q75) Have you ever had an overnight stay in a hospital for suspected or diagnosed COVID-19?
- ○ Yes
- ○ No

Q76) Which of the following occurred as a result of your symptoms? *Select all that apply*.

- I was kept overnight in a hospital because a healthcare provider thought I had COVID-19
- I saw a healthcare provider in person, such as in a clinic, doctor's office, urgent care, or Emergency Room (ER)/Emergency Department (ED)
- I spoke to a healthcare provider over the phone, by email, or online
- I self-isolated or quarantined at home
- None of the above

Q77) How likely are you to get vaccinated for COVID-19 once a vaccination is available to the public?

- Very unlikely
- Somewhat unlikely
- Somewhat likely
- Very likely
- Unsure

The following questions ask about behaviors to stop the spread of COVID-19. For each question, please indicate how often you do these activities.

Q78) How often do you wear a face mask or cover your face with a scarf, bandana, etc. when in public spaces?

- Not at all
- Some of the time
- Much of the time
- All of the time

Q79) How often do you engage in "social distancing" (keeping at least 6 feet away from other people) when in public places?

- Not at all
- Some of the time
- Much of the time
- All the time

Q80) Have you isolated yourself due to known or suspected exposure to COVID-19?

- Yes, I have isolated myself
- No, I had wanted to isolate myself but couldn't
- No, I didn't want to isolate myself when I had known or suspected exposure to COVID-19
- No, I never had known or suspected exposure to the COVID-19

Q81) What year were you born? ____

- I don't know
- Decline to state

Q82) Only answer this question if you selected "**I don't know**" for Q81.
About how old are you?____

Q83) What country were you born in? ____

Q84) Only answer this question if you did not write "**United States**" for Q83: About how long have you been in the United States? *Round to the nearest year.*

____

<u>Q85)</u> What term(s) best expresses how you describe your gender identity? S*elect all that apply*.
- o Man
- o Woman
- o Non-binary
- o Transgender
- o Other; please specify: ______________________________________

<u>Q86)</u> What is the highest degree or level of school you have completed, inside or outside the US? *Select only one answer*.
- o Less than 9th grade
- o Some high school
- o High school graduate or equivalent (GED)
- o Technical, trade, or vocational
- o Some college, but degree not received or is in progress
- o Associate degree (AA, AS)
- o Bachelor's degree (BA, BS, AB)
- o Graduate degree (Master's, Professional, Doctorate)

<u>Q87)</u> What is your marital status? *Select only one answer.*
- o Married or with a partner
- o Widowed
- o Divorced
- o Separated
- o Single

<u>Q88)</u> How many total people (adults and children) **currently** live in your household, including yourself? *Please enter a number.* ____

<u>Q89)</u> How many people under 18 years-old **currently** live in your household? *Please enter a number*.

____

<u>Q90)</u> How many people aged 65 or older **currently** live in your household? *Please enter a number*.

____

<u>Q91)</u> Are **you** an ESSENTIAL WORKER (e.g., healthcare, delivery worker, store worker, security, building maintenance)?
- o Yes
- o No
- o Unsure

<u>Q92)</u> Only answer this question if you selected "**Yes**" for Q91: Are **you** a first responder, healthcare provider, or other worker in a facility treating COVID-19?
- o Yes
- o No

<u>Q93)</u> Are **any other adults living in the home** an ESSENTIAL WORKER (e.g., healthcare, delivery worker, store worker, security, building maintenance)?
- o Yes
- o No
- o There is no other adult living in my home besides me

Q94) Only answer this question if you selected "**Yes**" for Q93: Are **they** a first responder, healthcare provider or other worker in a facility treating COVID-19?

- ○ Yes
- ○ No

Q95) In 2019 what was your total household income before taxes? *Select only one answer.*

- ○ Less than $5,000
- ○ $5,000 - $11,999
- ○ $12,000 - $24,999
- ○ 25,000 - $34,999
- ○ $35,000 - $49,999
- ○ $50,000 - $74,999
- ○ $75,000 - $99,999
- ○ $100,000 - $149,999
- ○ $150,000 - $199,999
- ○ $200,000 and above

Q96) Which one applies to you? *Your information will only be used for statistical analyses conducted by the research team and will not be used for any other purpose or shared with any other parties.*

- ○ US citizen
- ○ Temporary resident/green card
- ○ Permanent resident/green card
- ○ Visa holder, (enter type of visa): ______________________________
- ○ Temporary Protected Status (TPS)
- ○ Deferred Action for Childhood Arrivals (DACA)
- ○ Refugee
- ○ Undetermined
- ○ Prefer not to answer
- ○ Other, please describe: ______________________________

Q97) What are the main ways you get information about the pandemic? *Select all that apply.*

- ○ Government health websites (like the CDC or Department of Health)
- ○ Searching for information online (like Google)
- ○ Social media (like Facebook, Instagram, and Twitter)
- ○ Television news or radio
- ○ Newspaper, magazines
- ○ Family/friends
- ○ Health care providers, like doctors
- ○ Community or faith leaders
- ○ Other, please specify: ______________________________
- ○ None of the above

Q98) How often do you get information about the pandemic?

- ○ Less than once a month
- ○ Once a month
- ○ Several times a month
- ○ Once a week
- ○ Several times a week
- ○ Once a day
- ○ 2-10 times a day
- ○ More than 10 times a day

Q99) How informed would you say you are about the current pandemic?

- ○ Not very informed
- ○ Somewhat informed
- ○ Very informed

Q100) Does information about COVID-19 ever make you feel anxious/overwhelmed?
- Yes - Often
- Yes - Sometimes
- No

Q101) We are interested in understanding geographic differences in experiences with COVID-19 pandemic. To help us analyze survey responses across the entire United States, please provide your **CURRENT 5-digit ZIP code (i.e., where you CURRENTLY live)** below. Your address information will only be used for statistical analyses conducted by the research team and will not be used for any other purpose or shared with any other parties.

____

Q102) How did you learn about this survey?
- Referred by someone who also took the survey
- Referred by an organization; Please specify the organization:

  ____
- Other; please specify: ______________________________________
